# The Use of Iliac Stem Prosthesis for Acetabular Defects following Resections for Periacetabular Tumors

**DOI:** 10.1155/2013/717031

**Published:** 2013-10-22

**Authors:** Massimiliano De Paolis, Alessio Biazzo, Carlo Romagnoli, Nikolin Alì, Sandro Giannini, Davide Maria Donati

**Affiliations:** Rizzoli Orthopaedic Institute, via Cesare Pupilli 1, 40136 Bologna, Italy

## Abstract

*Introduction*. The management of pelvic tumors is a challenge for orthopaedic oncologists due to the complex anatomy of the pelvis and the need to have extensive exposure. Various reconstructive techniques have been proposed with poor functional results and a high percentage of complications. Our purpose is to determine the functional results and the rate of complications of iliac stem prosthesis for acetabular defects following resections for periacetabular tumors. *Materials and Methods*. Between 1999 and 2012, 45 patients underwent pelvic resections for periacetabular bone tumors followed by reconstruction with stem cup prosthesis. The most common diagnosis was CS (chondrosarcoma, 29 cases), followed by OS (osteosarcoma, 9 cases) and metastasis (3 cases). In 33 cases, this implant was associated with massive bone allografts. Minimum follow-up required to evaluate functional outcome was 2 years. We classified pelvic resections according to Enneking and Dunham's classification and we used MSTS (musculoskeletal tumor system) score to evaluate functional outcomes. *Results and Discussion*. Sixteen patients died of their disease, three were lost to follow-up, four are alive with disease, and twenty-two are alive with no evidence of disease. Fifteen patients had local recurrence. Sixteen patients had bone or lung metastasis. We have had 6 infections, 2 aseptic loosening, and 2 cases of hip dislocation. Iliac sovracetabular osteotomy was fused in all cases at 10 months from surgery. Functional results were good or excellent in 25 of 31 patients with long-term follow-up (77%), with a percentage similar to that reported in the literature. *Conclusion*. The use of iliac stem prosthesis is a simple reconstructive technique that reduces operative times and risk of infection. It allows having good results and low rate of complications, but it should be performed in selected cases and centres of reference.

## 1. Introduction

The management of pelvic tumors is a challenge for orthopaedic oncologists. Since 1980, when Enneking and Dunham [1] described the first classification of pelvic resections, internal hemipelvectomy has gradually replaced hindquarter amputation due to the improvements of surgical techniques and chemotherapy. Because the anatomy of the pelvis is complex, extensive exposure is needed to identify and protect major neurovascular structures.

This surgery is characterized by long operative times and consequently high number of complications, as reported by several authors [2–10].

Various reconstructive techniques have been proposed, such as iliofemoral coaptation and ischiofemoral arthrodesis, with poor functional results. The use of saddle or custom-made prosthesis can improve hip function in major resections but is characterized by high number of complications.

The use of large pelvic allograft is the most common technique to restore acetabular defects, but this procedure may be associated with high rate of complications, such as infections, nonunions, and fractures.

We believe that the use of iliac stem prosthesis, alone or in association with pelvic allograft, for acetabular defects following resections for periacetabular tumors simplifies reconstructive techniques and consequently can reduce the complications.

This technique can be used in a wide variety of patients, including children and patients who have received chemotherapy.

Our purpose is to determine the functional results and the rate of major complications of this type of reconstructions.

## 2. Materials and Methods

**Figure 1:**
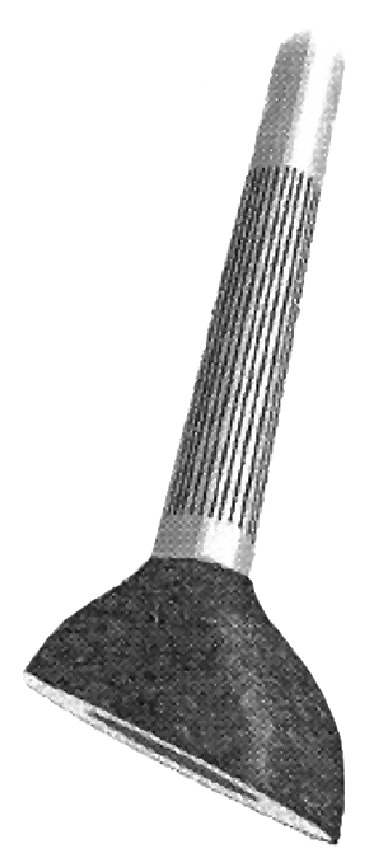
Iliac stem prosthesis.

Between 1999 and 2012, 45 patients underwent pelvic resections for periacetabular bone tumors followed by reconstruction with stem cup prosthesis (Figure 1). In 33 cases, this implant was associated with massive bone allografts, the so-called composite iliac stem prosthesis (CISP): stem cup prosthesis combined with allograft. The minimum follow-up required to evaluate functional outcome was 2 years.

General data and oncological outcomes are recorded (Table 1). Twenty-four men and 21 women were enrolled in the study. The average age was 47 years (range 17–79 yrs). The mean follow-up was 60 months (range 1–154 months). The most common diagnosis was CS (chondrosarcoma, 29 cases), followed by OS (osteosarcoma, 9 cases) and metastasis (3 cases).

The patients underwent staging exams according to Enneking's stage system [11]. The most common stage was IIB.

All patients received intravenous antibiotic therapy preoperatively and for 3 weeks after surgery. When the tumor was located in the periacetabular region, an ilioinguinal surgical approach was used with an anterolateral and ileofemoral extension in order to obtain a good view of the neurovascular structures and a good internal and external exposure of the pelvis and hip joint. When the tumor was located in the proximal femur, an extended ileo-femoral approach was performed.

**Figure 2:**
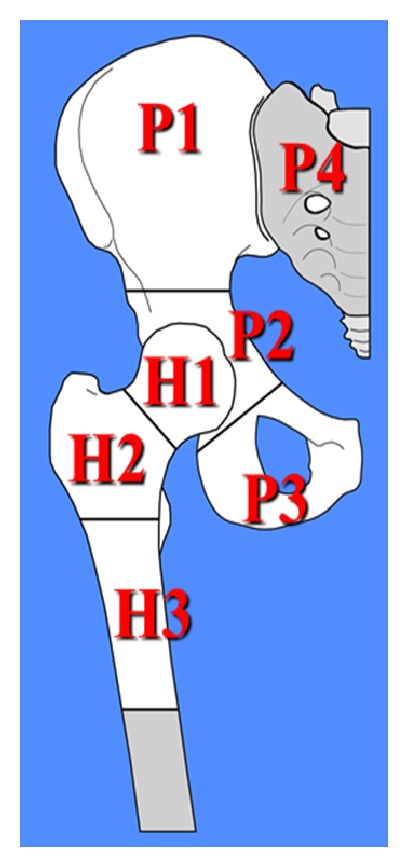
Pelvic and proximal femoral resections according to Enneking and Dunham's classification.

We classified pelvic resections according to Enneking and Dunham's classification [1] (Figure 2).

We performed 13 extra-articular femoral resections, removing the entire hip en bloc, when the tumor was located in the proximal femur and involved the joint and 32 intra-articular acetabular resections when the tumor was located in the pelvis, all of them involving P2 area. The most common type of resection was P2-P3 (14 cases) followed by P2 (12 cases). The mean operative time was 5.9 hours (range 4–10 hours). Complications and functional outcome are reported (Table 2).

**Figure 3:**
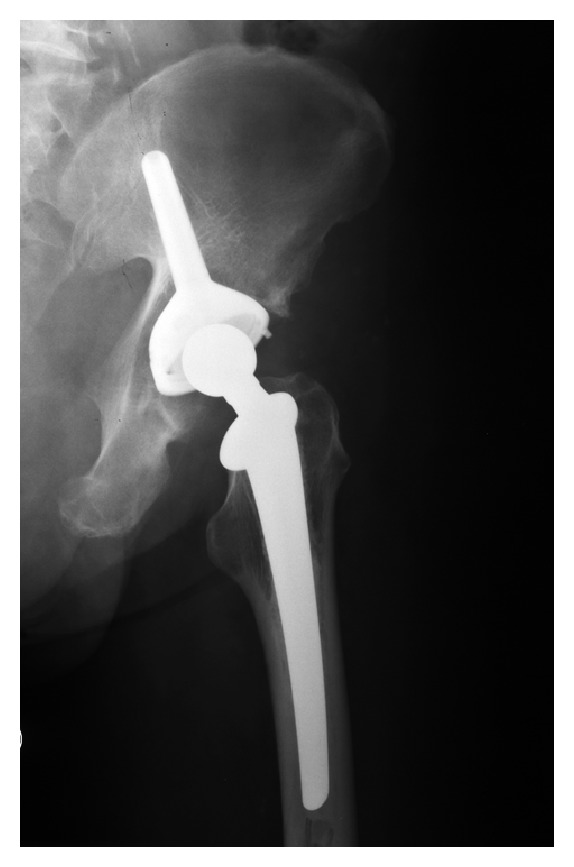
Reconstruction only with iliac stem prosthesis.

**Figure 4:**
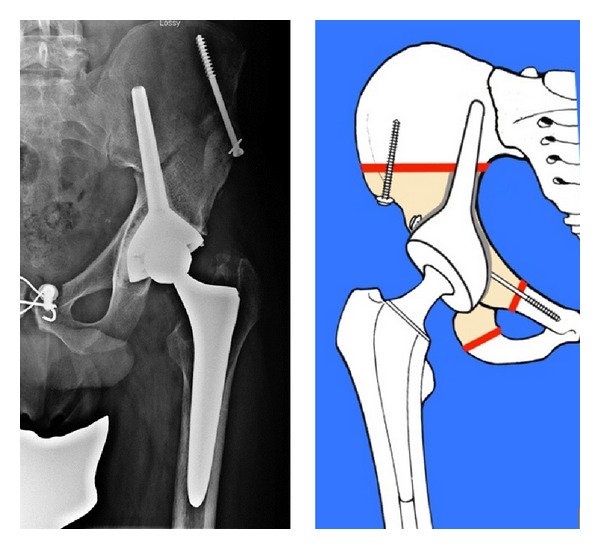
Reconstruction with CISP (P2 resection).

Reconstructions were performed all with iliac stem prosthesis (Figure 3) and in 33 cases associated with massive nonirradiated pelvic allograft stored at −80°C and thawed in rifampin solution (Figure 4).

In partial P2 resection, if residual bone stock was sufficient to obtain a stable implant, only stem cup prosthesis was used.

In P2 or P2-P3 resections we used CISP: stem prosthesis cemented in the allograft and press-fit in the residual iliac host bone (Figure 4).

**Figure 5:**
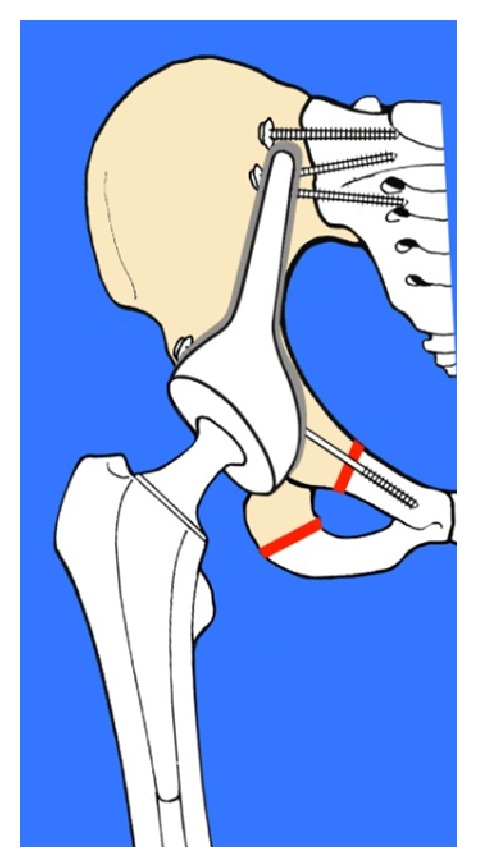
Reconstruction with CISP (P1-P2 resection).

In P1-P2 resections, the stem cap prosthesis was completely cemented (Figure 5).

In cases of CISP is very important to obtain a good matching between graft and host bone. Moreover, to improve the donor-to-host bone contact, a transiliac screw is added (Figures 4 and 8). Either screws or a cerclage wire can be used for osteosynthesis of the anterior pelvic arch (Figure 4). This technique does not include the use of reconstruction plates along the innominate line or compression plates across the iliac osteotomy line. Occasionally, in order to improve implant stability, an artificial ligament is fixed between the ileopubic branch and the intertrochanteric region of the femur.

Hemovac drains were removed at a mean of 4-5 days after surgery. Partial weight bearing was started at 6 months and total weight bearing was begun at 9–12 months. Hip cast was used for the first 3 months.

Patients had follow-ups with X-ray of the pelvis and CT of the chest every 3 months during the first 3 years, then every 6 months during the fourth and fifth year, and yearly thereafter. Radiographic evaluation was assessed by two authors (DD and MD), in order to evaluate signs of nonunion, resorption of the graft, failure, or migration of the prosthetic components. Union of the graft to the host bone was said to have occurred if there was no visible osteotomy line at the junction sites or if greater than or equal to 75% of cortical thickness was fused on follow-up radiographs, according to the ISOLS radiological implants evaluation system [12]. Margins were classified as intralesional, marginal, and wide. Margins were wide in 31 cases, marginal in 10 cases, and intralesional in 2 cases.

Functional evaluation was performed at the most recent follow-up examination and using MSTS score system [13].

**Figure 6:**
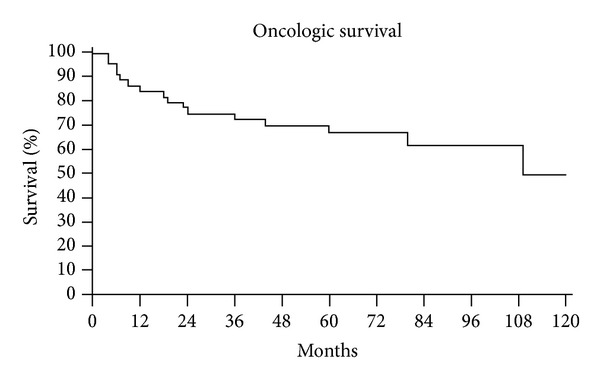
Kaplan-Meier patient survival curve.

**Figure 7:**
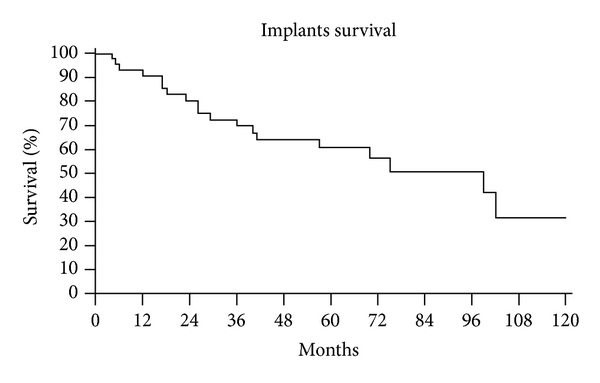
Kaplan-Meier implant survival curve.

Patients' (Figure 6) and prosthesis survival (Figure 7) was determined using Kaplan-Meier analysis. The end point for prosthetic survival was amputation or prosthesis removal.

The study was approved by the Institutional Ethics Committee and patients were informed about the proposed surgical procedure and alternative procedures, and informed consent was obtained in each case.

### 2.1. Oncological Results

Sixteen patients died of their disease, three were lost to follow-up, four are alive with disease, and twenty-two are alive with no evidence of disease.

Fifteen patients had local recurrence (33%): margins were wide only in 9 of them. Ten were CS. All of them were extracompartimental, stage IB, or IIB.

Six of them were treated with hindquarter amputation, three with chemotherapy and radiotherapy, three with excision of the local recurrence, and one with resection and reconstruction with saddle prosthesis. One refused hindquarter amputation; one was lost to follow-up. Sixteen patients had bone or lung metastasis.

### 2.2. Complications and Functional Results

The probability to have the prosthesis still in place was 60% at medium follow-up of 5 years (Figure 7).

We have had 6 infections (13%) that required in case 15 and 44 allograft and prosthesis removal (treated, resp., with cement spacer and new osteosynthesis with plate—case 15—and with modular prosthesis revision—case 44—), in 1 case only allograft removal (case 11—replaced with cement spacer), in 1 case surgical debridement (case 36), and in two cases hindquarter amputation (case 22 and 26). Three of them were patients who underwent chemotherapy for osteosarcoma.

Case 11 was a IIB OS that received chemotherapy and extrarticular femoral resection (H1-H2-P2) and reconstruction with allograft prosthetic composite (APC, i.e., femoral revision stem and femoral allograft). At last follow-up (99 months) he has no evidence of disease and poor function. Case 15 was a wide resection (P1-P2-P3) for a central CHS staged IB. A composite reconstruction was performed, but he developed lung metastasis and at last follow-up (75 months) he is alive with disease. Case 22 was a central CHS staged IB and treated with P2 resection and reconstruction with CISP. At last follow-up (112 months) she has no evidence of disease. Case 26 was a central CHS staged IB and treated with P2-P3 resection and reconstruction with CISP. She was lost to follow-up at 5 months. Case 36 was a IIB angiosarcoma who underwent P2-P3 resection and reconstruction with CISP. She was lost to follow-up at 38 months. Case 44 was a IIB OS that underwent P1-P2-P3 resection and reconstruction with CISP. At last follow-up she has a poor function.

We have had 2 aseptic loosenings (case 1 and 9), due, respectively, to allograft fracture during operation and wrong positioning of the stem cup prosthesis.

Case 1 was a P2 resection and was treated with revision of the implant (allograft and prosthesis). At last follow up (144 months) he has no evidence of disease and good function.

Case 9 was a P2 resection reconstructed only with stem cup prosthesis and underwent revision of the prosthesis. At 127 months from surgery she has no evidence of disease and fair function.

We report 2 cases of hip dislocation (case 12 and 27) both treated with revision surgery using longer head prosthesis and an artificial ligament between iliopubic branch and intertrochanteric area of the femur. Case 12 was a P2 resection and it has no evidence of disease at last follow-up (84 months) and has an excellent function. Case 27 was a radio-induced OS treated with P2-P3 resection and CSIP. She developed local recurrence, but she refused amputation.

Among minor complications, we report 2 cases of sciatic popliteal nerve palsy, partially recuperated, 2 cases of delay healing of wound surgery, 3 vein thrombosis, and 1 intolerance to sacroiliac screw which was removed.

**Figure 8:**
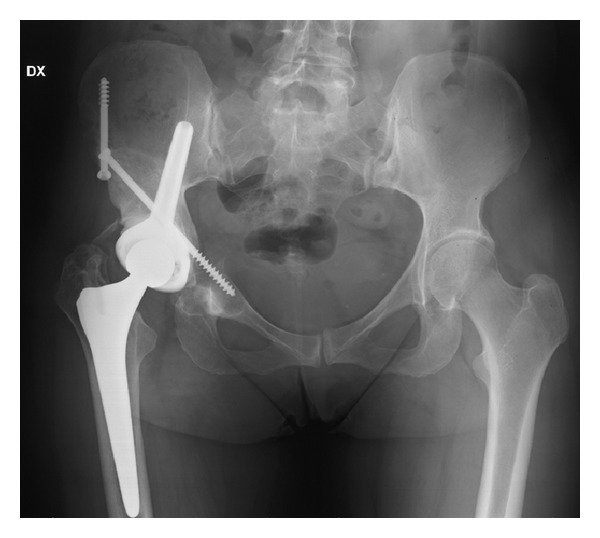
Nonunion of the anterior pelvic ring in a P2 resection and reconstruction with CISP.

Iliac sovracetabular osteotomy was fused in all cases at 10 months from surgery, while we report a frequent nonunion of anterior pelvic ring that has not affected implant stability (Figure 8).

Three of the patients evaluated with long follow-up had a resorption at the prosthesis-host bone interface, with a migration of the implant <20 mm in 2 of them. Anyone underwent revision surgery.

Functional results were good or excellent in 25 of 31 patients with long-term follow-up (77%). Sixteen of them were partial P2 resections or P2-P3 resections reconstructed only with iliac stem prosthesis.

## 3. Discussion

Surgical management of periacetabular tumors is a challenge for orthopaedic surgeons because of the complex anatomy of the pelvis and because there are important anatomical structures to preserve. Pelvic continuity and durable hip function are very difficult to achieve.

Several reconstructive options have been described to restore pelvic ring continuity, such as saddle prosthesis, custom-made prosthesis, and the use of allografts, alone or in association with prosthesis (composite reconstruction).

Saddle prosthesis was first described by Nieder in 1979 to fill wide acetabular defects in revision hip surgery. Then, they were used to reconstruct pelvic anatomy following resections for periacetabular tumors. But several studies [5, 8, 14, 15] evaluated this type of reconstruction reporting all limitations, including inacceptable functional results and high rate of complications, so their use is reserved to salvage surgery.

One of the first studies dealing with custom-made prosthesis was performed by Abudu et al. [16] in 1997. They analyzed the results of periacetabular resections performed on 35 patients for primary malignant tumors of the pelvis. According to them, the most important advantage of this technique was the possibility of a personalized reconstruction, which means to make the prosthesis preoperatively. They reported good functional results but high number of complications, above all infections and dislocations (60%), that required revision surgery in 40% of cases.

Customized prosthesis is made based on preoperative imaging data, which may not reflect the situation after resection if tumor-related or mechanical problems are discovered at surgery and require more extensive resection than planned. Conversely, pelvic allografts are larger than needed and are cut to size at the end of the resection step [3].

The most important advantages of composite reconstructions are the possibility to restore any bone defect by fitting the graft during surgery as well as osteointegration, revascularization, and partial replacement with host bone and the possibility to suture soft tissues to the graft, providing better results by developing a biologic union with the recipient bone. The most important disadvantage is the high infection rate, reported between 10 and 50% in the literature. Other disadvantages are fracture, non-union, the risk of viral disease transmission, and the necessity to have a musculoskeletal tissue bank.

Few records are reported in the literature about the use and results of pelvic allografts. In 1996, Ozaki et al. [2] presented the outcomes of 22 pelvic reconstructions with massime allografts for pelvic sarcomas, reporting high complication rates, such as 2 allograft fractures and 8 infections. Nine of them were removed. In 2001, Langlais et al. [3] reported the long-term results of hemipelvis reconstructions with allografts in 13 patients. Major complications were 1 infection and 2 dislocations. Functional results were good in 56% of patients.

In 2000, Yoshida et al. [17] described the outcomes of 19 patients who underwent reconstruction with pelvic allografts for periacetabular sarcomas. More frequent complications were 1 allograft fracture and 4 migrations of the bipolar prosthesis, which had led to total hip arthroplasty in 2 of them.

Delloye et al. [6] described the results of 18 reconstructions with allograft prosthetic composite in patients treated for sarcoma of the periacetabular region, reporting good results with rates of infection and functionality of 12.5% and 73%, respectively. This positive progression can be partly justified by the author by improving surgical techniques and imaging and the use of rifampin with which they thawed grafts. Best results (82% MSTS score) were obtained in children and adolescents. However, the reported rate of local recurrence was 29%, comparable to our percentage rate of 31%.

More recently, Donati et al. [7] analyzed the results of three different reconstructive techniques for periacetabular sarcomas. They divided patients in to three groups: the last of them was made up of 5 patients who underwent pelvic composite reconstruction with massive allografts with the same iliac stem prostheses described in this study. None of them developed mechanical complications or infections. This is the unique report about this type of reconstruction described in the literature.

Our purpose in this study was to determine implant survival and functional outcomes of these patients.

The most important limitation of our study is the presence of many uncontrolled variables, such as tumor grade and extent, the treatment, patient age, and reconstructive procedure, which have a confounding effect on dependent variables such as prosthesis survival. Moreover, there is the difficulty to compare our results with those of other authors because of differences in tumor type, extent of resection, and reconstruction techniques.

We observed 6 infections (13%), a rate similar to other series. All of them occurred in patients who underwent composite reconstruction with massive allografts and 3 of them in patients who underwent chemotherapy for OS. Other series, like for example, Ozaki's one [2], reported infection rate of 30%, which can be explained according the author to the long operative time, large blood loss, chemotherapy, and radiation therapy. In his series, infection was commoner in CS than in OS or ES, in large tumors than in small tumors, after a long operation time than after a short one. Indeed, the average tumor volume of CS was significantly larger than that of ES or OS, because chemotherapy reduces tumour volume before surgery: this may reduce operative times and consequently infection rate.

The use of rifampin solution to thaw the allografts is a factor influencing our low rate of infections, but other factors must be searched to explain it. According to us, the most important factor reducing infections is the low operative time (mean time 5.9 hours), due to an easy surgical technique that avoids the use of long plates and in selected cases the use of the stem cup prosthesis without allograft, such as when resection line can spare part of the acetabular roof and posterior wall.

We reported 2 aseptic loosening of the implant, due to wrong positioning of the prosthesis during surgery (that leads to an improper usury of polyethylene) and to an intraoperative fracture of the allograft.

Two dislocations are reported: both cases were not treated with artificial ligament. They were revised with longer head prosthesis and an artificial ligament between iliopubic branch and intertrocanteric area of the femur.

We had 15 local recurrences (30%). Among these patients 6 had inadequate margins and 8 were high-grade sarcomas. Ten were CS and all were extracompartimental, stage IB, or IIB. This high rate of local recurrence can be justified by different variables, such as the inadequate margins of resection in 6 of them (Donati et al. [4] have demonstrated yet that margins have a significant effect on the rate of local recurrence of CS); the huge volume of these tumors (indeed, tumors that usually occur in the pelvis are diagnosed late and therefore are of large size); the fact that chemotherapy has no effect on CS.

Our functional results are similar to those reported in the literature, between 56% and 75%.

## 4. Conclusions

The use of a stemmed acetabular cup, alone or in association with a massive bone allograft, seems to be a valid alternative and a simple reconstruction technique. This type of reconstruction avoids complex osteosynthesis, such as contoured plates, therefore reducing significantly the operative time and early complication rates.

It allows having good results and low rate of complications, but it should be performed in selected cases and centres of reference.

## Figures and Tables

**Table 1:** General data.

Patient	Gender	Age (years)	Stage	Diagnosis	Margins	Chemotherapy	Local recurrence	Metastasis	Status	Patient follow-up (months)
1	M	38	IA	Central CHS	Wide	No	No	No	NED	144
2	W	48	IIB	CHS dediff	Wide	Yes	Yes	Yes	DOD	44
3	M	40	IIA	Central CHS	Wide	No	No	No	NED	74
4	W	37	III	Meta OS	Wide	Yes	No	Yes	DOD	24
5	W	44	IIB	Central CHS	Wide	No	Yes	Yes	DOD	109
6	W	71	IIB	CHS	Wide	No	No	Yes	DOD	7
7	W	26	IIB	OS	Wide	Yes	No	No	DOD	60
8	W	22	/	Mec failure	/	No	No	No	NED	154
9	W	23	IB	Perif CHS	Marginal	No	No	No	NED	127
10	W	68	IB	Central CHS	Marginal	No	Yes	No	DOD	80
11	M	17	IIB	OS	Wide	Yes	No	No	NED	99
12	M	48	IB	Central CHS	Wide	No	No	No	NED	84
13	M	50	IB	Perif CHS	Intralesional	No	No	No	NED	103
14	W	69	IB	Central CHS	Wide	No	No	No	NED	95
15	M	37	IB	Central CHS	Marginal	No	No	Yes	AWD	75
16	M	26	IIB	Meta ES	Intralesional	Yes	Yes	Yes	DOD	24
17	M	70	IB	Central CHS	Wide	No	No	No	NED	94
18	M	52	IB	CHS dediff	Wide	No	Yes	No	DOD	26
19	M	27	III	OS	Wide	Yes	Yes	Yes	DOD	36
20	M	32	IB	Central CHS	Wide	No	No	No	NED	104
21	W	43	IB	OS	Wide	No	No	No	NED	77
22	W	59	IB	Central CHS	Wide	No	Yes	No	NED	112
23	M	27	/	Mec Failure	/	Yes	No	No	NED	101
24	M	75	III	Meta K	Wide	No	No	No	LOST	10
25	W	78	IIB	Central CHS	Wide	No	No	Yes	DOD	7
26	W	58	IB	Central CHS	Wide	No	Yes	No	LOST	5
27	W	79	IIB	OS Rx-induced	Marginal	No	Yes	No	AWD	41
28	W	39	IB	Central CHS	Marginal	yes	Yes	Yes	AWD	44
29	M	36	III	OS	Wide	yes	Yes	Yes	DOD	11
30	M	60	IB	Central CHS	Wide	No	No	No	NED	70
31	M	23	III	OS	Wide	Yes	No	Yes	DOD	24
32	M	56	IB	Central CHS	Wide	no	No	Yes	DOD	1
33	W	36	IIB	OS	Wide	Yes	Yes	No	DOD	14
34	M	60	IB	Central CHS	Wide	No	No	No	NED	67
35	M	65	IB	Central CHS	Wide	No	Yes	Yes	AWD	68
36	W	63	IIB	Angiosarc.	Wide	yes	No	Yes	LOST	38
37	M	18	IIB	ES	Marginal	Yes	No	No	NED	72
38	W	45	IIB	CHS dediff	Wide	yes	Yes	Yes	DOD	4
39	M	56	IB	Central CHS	Wide	No	No	No	NED	58
40	W	57	IB	Central CHS	Wide	No	No	No	NED	68
41	M	63	IA	Central CHS	Wide	No	No	No	NED	55
42	M	51	IB	Central CHS	Marginal	No	No	No	NED	58
43	M	66	IB	Central CHS	Marginal	No	No	No	NED	64
44	W	33	IIB	OS	Wide	Yes	No	No	NED	58
45	W	62	IB	Central CHS	Marginal	No	Yes	Yes	DOD	40

M: man; W: woman; CHS: chondrosarcoma; OS: osteosarcoma; ES: Ewing sarcoma; K: carcinoma; NED: no evidence of disease; DOD: dead of disease; AWD: alive with disease.

**Table 2:** Type of resection, operative time, complications, and functional outcome of the patients. Artificial ligament was used in 21 cases.

Patient	Type of resection	Surgical time (hours)	Artificial ligament	Allograft	Infection	Joint instability	Fracture	Nonunion	Mechanical failure	Implant survival (months)	MSTS score
1	P2-P3	6	Yes	Yes	No	No	Yes	No	Yes	103	Good
2	P2-P3	6	Yes	Yes	No	No	No	No	No	29	Fair
3	P2-P1	7	Yes	Yes	No	No	No	No	No	74	Good
4	P1-P2	5	Yes	Yes	No	No	No	No	No	24	Good
5	P2	6,5	Yes	Yes	No	No	No	No	No	72	Good
6	H1-H2-P2	7,5	No	No	No	No	No	No	No	7	Not evaluated
7	H1-H2-P2	5	No	Yes	No	No	No	No	No	60	Excellent
8	H1-H2-P2	4	Yes	Yes	No	No	No	No	No	154	Good
9	P2-P3	6,5	No	No	No	No	No	No	Yes	35	Fair
10	P2-P3	/	No	No	No	No	No	No	No	26	Fair
11	H1-H2-P2	5	No	Yes	Yes	No	No	No	No	19	Fair
12	P2	5,5	No	Yes	No	Yes	No	No	No	55	Excellent
13	H1-P2	7	No	Yes	No	No	No	No	No	103	Good
14	H1-P2	6	No	Yes	No	No	No	No	No	95	Excellent
15	H1-P1-P2	7	Yes	Yes	Yes	No	No	No	No	8	Not evaluated
16	P-P3	8	Yes	No	No	No	No	No	No	21	Not evaluated
17	P2	5	No	No	No	No	No	No	No	94	Excellent
18	P2	7	Yes	Yes	No	No	No	No	No	27	Good
19	H1-H2-P2-P3	6	No	No	No	No	No	No	No	26	Good
20	P2-P3	8	Yes	Yes	No	No	No	No	No	104	Excellent
21	P2	4	No	Yes	No	No	No	No	No	77	Excellent
22	P2	5	No	Yes	Yes	No	No	No	No	40	Fair
23	H1-P2	5	No	No	No	No	No	No	No	101	Good
24	P2-P3	6	No	No	No	No	No	No	No	10	Not evaluated
25	P1-P2	5	Yes	Yes	No	No	No	No	No	7	Not evaluated
26	P2-P3	4,5	Yes	Yes	Yes	No	No	No	No	7	Not evaluated
27	P2-P3	6,5	No	No	No	Yes	No	No	No	41	Excellent
28	P1-P2-P3	6	Yes	Yes	No	No	No	No	No	44	Good
29	P2-P3	6	No	Yes	No	No	No	No	No	6	Not evaluated
30	P2-P3	6	Yes	Yes	No	No	No	No	No	69	Excellent
31	H1-H2-P1-P2	6	No	Yes	No	No	No	No	No	24	Good
32	H1-P2-P3	6	Yes	Yes	No	No	No	No	No	1	Not evaluated
33	P1-P2	5	Yes	Yes	No	No	No	No	No	12	Not evaluated
34	P1-P2-P3	10	Yes	Yes	No	No	No	No	No	67	Good
35	P2-P3	6	No	Yes	No	No	No	No	No	17	Not evaluated
36	P1-P2	6	No	Yes	Yes	No	No	No	No	38	Poor
37	P2	6	Yes	Yes	No	No	No	No	No	72	Excellent
38	H1-P2-P3	6	No	No	No	No	No	No	No	4	Not evaluated
39	P2	5	Yes	No	No	No	No	No	No	58	Excellent
40	P2-P3	5	No	No	No	No	No	No	No	68	Poor
41	P2	5	No	Yes	No	No	No	No	No	55	Good
42	P2-P3	6	Yes	Yes	No	No	No	No	No	58	Good
43	P2	5	Yes	Yes	No	No	No	No	No	64	Excellent
44	P1-P2-P3	6	No	Yes	Yes	No	No	No	No	15	Not evaluated
45	H1-H2-H3-P2-P3	6	/	Yes	No	No	No	No	No	10	Not evaluated
